# Successional dynamics of marine fouling hydroids (Cnidaria: Hydrozoa) at a finfish aquaculture facility in the Mediterranean Sea

**DOI:** 10.1371/journal.pone.0195352

**Published:** 2018-04-02

**Authors:** Luis Martell, Roberta Bracale, Steven A. Carrion, Jennifer E. Purcell, Marco Lezzi, Cinzia Gravili, Stefano Piraino, Ferdinando Boero

**Affiliations:** 1 University Museum of Bergen, Department of Natural History, University of Bergen, Bergen, Norway; 2 Dipartimento di Scienze e Tecnologie Biologiche e Ambientali, Università del Salento, Lecce, Italy; 3 University of Central Florida, Orlando, Florida, United States of America; 4 Western Washington University, Bellingham, Washington, United States of America; 5 CoNISMa, Consorzio Nazionale Interuniversitario per le Scienze del Mare, Rome, Italy; 6 CNR-ISMAR, Istituto di Scienze Marine del Consiglio Nazionale delle Ricerche, UO Genova, Genoa, Italy; Evergreen State College, UNITED STATES

## Abstract

Aquaculture is increasing rapidly to meet global seafood demand. Some hydroid populations have been linked to mortality and health issues in finfish and shellfish, but their dynamics in and around aquaculture farms remain understudied. In the present work, two experiments, each with 36 panels, tested colonization (factors: depth, season of immersion) and succession (factors: depth, submersion duration) over one year. Hydroid surface cover was estimated for each species, and data were analyzed with multivariate techniques. The assemblage of hydrozoans was species-poor, although species richness, frequency and abundance increased with time, paralleling the overall increase in structural complexity of fouling assemblages. Submersion duration and season of immersion were particularly important in determining the species composition of the assemblages in the succession and colonization experiments, respectively. Production of water-borne propagules, including medusae, from the hydroids was observed from locally abundant colonies, among them the well-known fouling species *Obelia dichotoma*, potentially representing a nuisance for cultured fish through contact-driven envenomations and gill disorders. The results illustrate the potential importance of fouling hydroids and their medusae to the health of organisms in the aquaculture industry.

## Introduction

Aquaculture is playing an increasing role in meeting the protein needs of the growing world population [1]. The development of new aquaculture facilities has led to an increase in submerged structures such as floats, ropes, cages and nets that inadvertently provide favourable substrates for fouling organisms [2]. These biofoulers greatly interfere with culture operations, produce significant economic impacts on marine aquaculture, and are widely recognized as one of the main problems faced by any aquaculture facility: the annual direct economic cost of controlling biofouling on aquaculture is estimated conservatively around 5–10% of the industry value [3].

Common detrimental effects of biofouling in aquaculture include significant increases in the weight and drag of submerged structures, reduction of water flow through the nets of the cages, which compromises the environmental quality for fish and shellfish in terms of oxygen concentration and food limitation, overgrowth of shellfish stocks, and skin and gill lesions and disease in fish [2, 4–6]. Current antifouling strategies have been unable to cope efficiently with fouling, and further research on the dynamics of colonization and succession of fouling organisms is needed in order to prevent and manage biofouling in aquaculture [7–8].

Hydrozoans are a common component of biofouling assemblages in aquaculture facilities. They are renowned for their diverse reproductive patterns, ranging from completely benthic life cycles to completely pelagic ones, with >700 species having a combination of benthic and pelagic stages [9]. The most familiar forms, attached hydroids and swimming medusae, occur in aquaculture facilities worldwide. Benthic hydroids have been linked to a wide array of negative effects on shellfish and finfish culture, including net occlusion, reduced water flow, smothering of shells, devaluation of final products, competition with target species for food and space, disruption of feeding and valve opening, direct lesions, and disease transmission [2, 5, 10]. The medusae and other planktonic propagules produced by some of these hydroids can equally cause skin and gill damage in farmed fish by their stinging cnidocytes and injectable venoms, making cultured fish prone to bacterial infections and increased mortality [11–14].

Many species of hydrozoans foul aquaculture facilities around the world, yet most scientific knowledge on hydroid fouling comes from studies on the large and highly damaging species belonging to the families Tubulariidae (mainly members of genus *Ectopleura*) and Campanulariidae [15–20]. Unfortunately, studies on biofouling usually put all hydrozoans in one category, thus preventing analysis of the hydroid fouling dynamics and medusa production at the population and species level. An exception to this is the thesis of Bosch-Belmar [13], which examined both the fouling hydroids attached to the nets of the fish pens and the small reproductive stages they produce that can enter the gill chambers of the fish damaging them with stinging capsules (nematocysts). The author detailed the composition, growth and reproductive periods of hydroid assemblages on fish pens, as well as the planktonic stages of hydrozoans and gill damage and mortality of fish at two aquaculture facilities along the Mediterranean coast of southern Spain. Production of the planktonic propagules increased beginning 5 months after net installation, while abundances of the hydrozoans peaked in spring and autumn and coincided with fish kills by planktonic propagules of *Ectopleura larynx* (Ellis & Solander, 1786) and gill damage by all hydrozoans in the water [13–14].

The objectives of the present work were to describe the colonization and succession of fouling hydrozoan assemblages on panels immersed beside an aquaculture facility at Taranto, Italy (Central Mediterranean Sea) to test the roles of seasonality, submersion duration, and depth in the structuring of the fouling hydroid assemblages and to identify the species of fouling hydrozoans potentially harmful to the farmed fish in the aquaculture facility. Two experiments tested colonization (factors: depth, season of immersion) and succession (factors: depth, submersion duration) throughout one year in the central Mediterranean Sea.

## Material and methods

### Study area

The study was conducted at an aquaculture facility that produces sea bass (*Dicentrarchus labrax* (Linnaeus, 1758)) and sea bream (*Sparus aurata* Linnaeus, 1758) located in an area of muddy and sandy bottoms at the eastern sector of the Mar Grande basin in the Gulf of Taranto, NW Ionian Sea (40° 25’ 46.1” N, 17° 14’ 23.7” E). No permits were required for the described study, which complied with all relevant regulations. Mar Grande, with about 36 km^2^ area and 42 m of maximum depth [21], is a partially-enclosed basin hosting a wide array of nautical, industrial, productive, and commercial activities. The waters around Taranto routinely receive urban and industrial waste, which together with the intense ship traffic, have contributed to the high environmental stress observed in this area for decades [22]. On the other hand, the region historically has been one of the most important sea farming areas in Italy for the production of mussels and other shellfish [23–24]. In recent years, several fish farms have begun operations in the basin, usually in combination with mussel farming.

No direct observation of negative effects involving hydrozoans were reported in the aquaculture facility during the course of this study, but mass mortalities associated with fouling hydroids and pelagic hydromedusae occurred at the same time along the coast of Spain [13–14], highlighting the need for prospective studies on hydroid dynamics on aquaculture cages in our area. Benthic hydroids were observed fouling several hard surfaces at the aquaculture facility throughout the duration of the study, but monitoring of their succession and colonization was exclusively on test panels, as described below. Temperature data in the vicinity of the studied facility obtained from the Italian National Institute for Environmental Protection and Research are included as supplementary material (S1 Table).

### Experimental set-up and laboratory work

**Fig 1:**
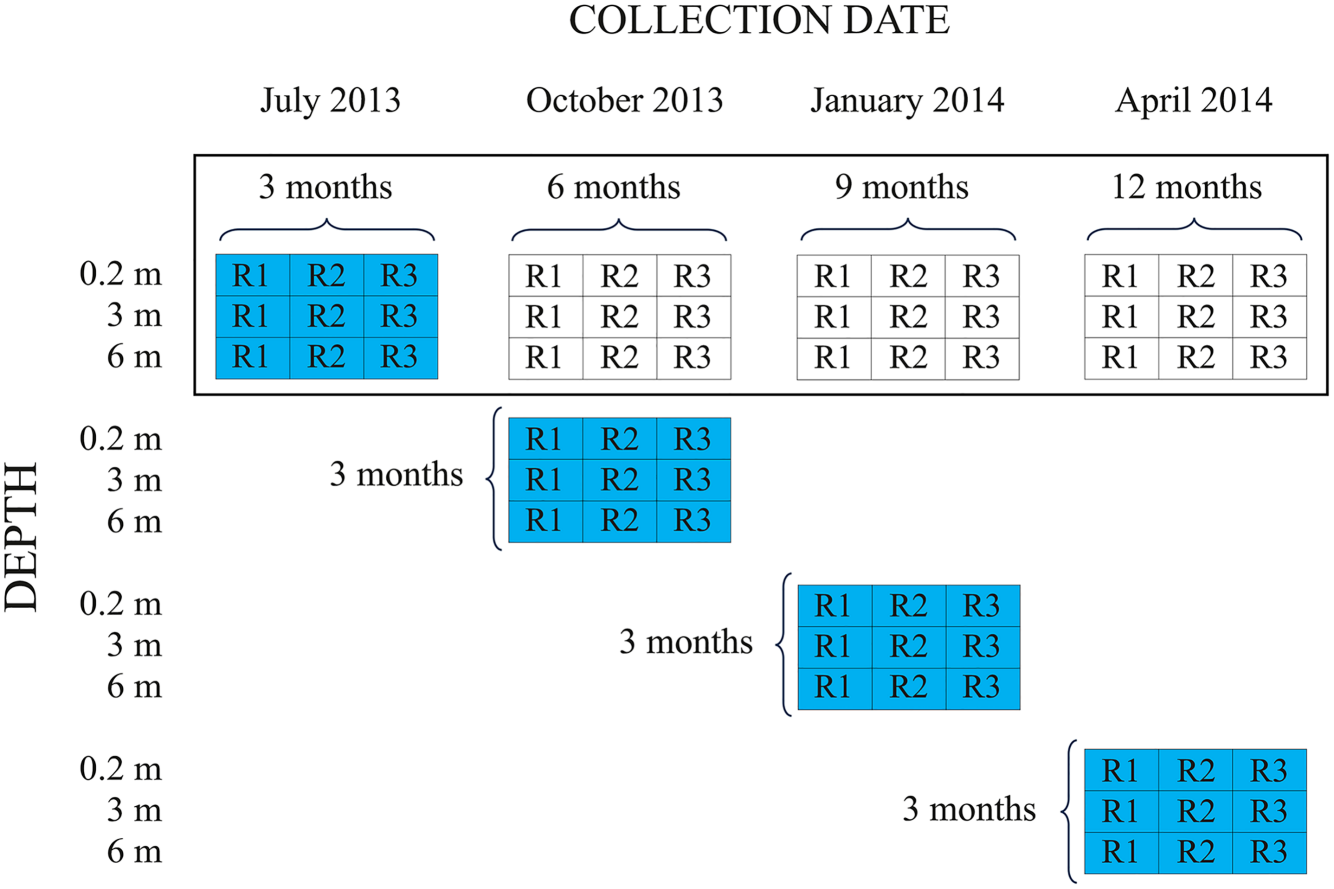
Experimental design to test hydroid succession and colonization at an aquaculture farm in the Central Mediterranean Sea (2013–2014). Three replicate test panels at each depth are identified as R1, R2, or R3. Panels for the succession experiment are inside the black rectangle and those for the colonization experiment are shaded in gray. The number of months that each set of panels spent underwater is indicated.

Two simultaneous experiments (one on succession, the other on colonization) began in April 2013. The fieldwork, sampling and laboratory methodologies were identical for both experiments. Different panels were analyzed in each experiment, except the first 9 panels deployed were the first set of panels analyzed in the succession experiment (submersion duration = 3 months) and in the colonization experiment (season of immersion = spring) (Fig 1). In the succession experiment, 36 roughened PVC panels (15 x 15 x 0.3 cm) were distributed on 12 vertical longlines that were deployed at 2–3 m intervals around a sea bream cage. Three panels were positioned on each longline at 0.2, 3, and 6 m depths. The total depth of the water column was ca. 10 m, while the cage extended to 9 m depth. Every three months, three of these longlines were detached and their respective panels taken to the laboratory for analysis. Thus, the succession panels were submerged for 3, 6, 9, or 12 months.

In the colonization experiment, 36 roughened PVC panels (15 x 15 x 0.3 cm) also were distributed on twelve vertical longlines deployed at 2–3 m intervals around a sea bream cage, but only three longlines were deployed and recovered in each sampling event. Thus, all analysed panels were submerged for a period of three months, having been deployed either in spring (April 2013), summer (July 2013), autumn (October 2013), or winter (January 2014).

The panels were collected, brought to the laboratory and photographed with a Nikon Coolpix E990 camera to estimate species coverage. Panels then were fixed in 4% formaldehyde for subsequent taxonomic analysis. All of the hydrozoans from the surface facing towards the cage of each panel were identified under 8x and 25x magnification of a stereomicroscope and photographed. No protected species were sampled during this study.

The hydrozoans were identified to species or the lowest possible taxonomic level following specialized literature [25–26]. Species richness (number of hydrozoan species per panel), the substrate on which each hydroid colony was growing, and the number of reproductive structures per species (gonothecae or medusa buds) were recorded. The total surface area covered by each hydrozoan species on each panel was calculated from the photographs with the image analysis software ImageJ [27]. The standardized area (per 100 cm^2^) was used as the estimate of total abundance for each species in subsequent analysis. For each species producing medusae or planktonic propagules, the number of reproductive structures per 100 cm^2^ also was calculated by directly counting either medusa buds, medusae developing inside gonothecae, or planktonic propagules. The substrate preference of the fouling hydroids as a whole was calculated as the percentage of occurrences per available substrate in each combination of depth and submersion duration in the succession experiment. All specimens were deposited in the Hydrozoa Collection of the University of Salento (Lecce, Italy) (S2 Table).

### Data analysis

Differences in the total abundance and species richness of the fouling hydroids on each panel were tested through a series of two-way Analysis of Variance (ANOVA). The factors tested were depth and submersion duration in the succession experiment, and depth and season of immersion in the colonization experiment. Multivariate analyses were used to compare the similarity of the assemblages of fouling hydroids on the panels according to the tested factors in each experiment. Non-metric multidimensional scaling (nMDS) analyses based on Bray-Curtis distances of square root-transformed surface area data (samples with no hydrozoans excluded) were performed to visualize changes in species assemblages. To test the differences in the composition of the assemblages in relation to factors ‘submersion duration’ in the succession experiment (fixed, four levels), ‘season of immersion’ in the colonization experiment (fixed, four levels), and depth in both experiments (fixed, three levels), distance-based permutational multivariate analysis of variance was used (PERMANOVA, [28]). Finally, the similarity percentage procedure SIMPER enabled calculation of the contribution of each species to the observed patterns. All multivariate analyses were performed using the PRIMER software package [29].

## Results

### Succession experiment

**Fig 2:**
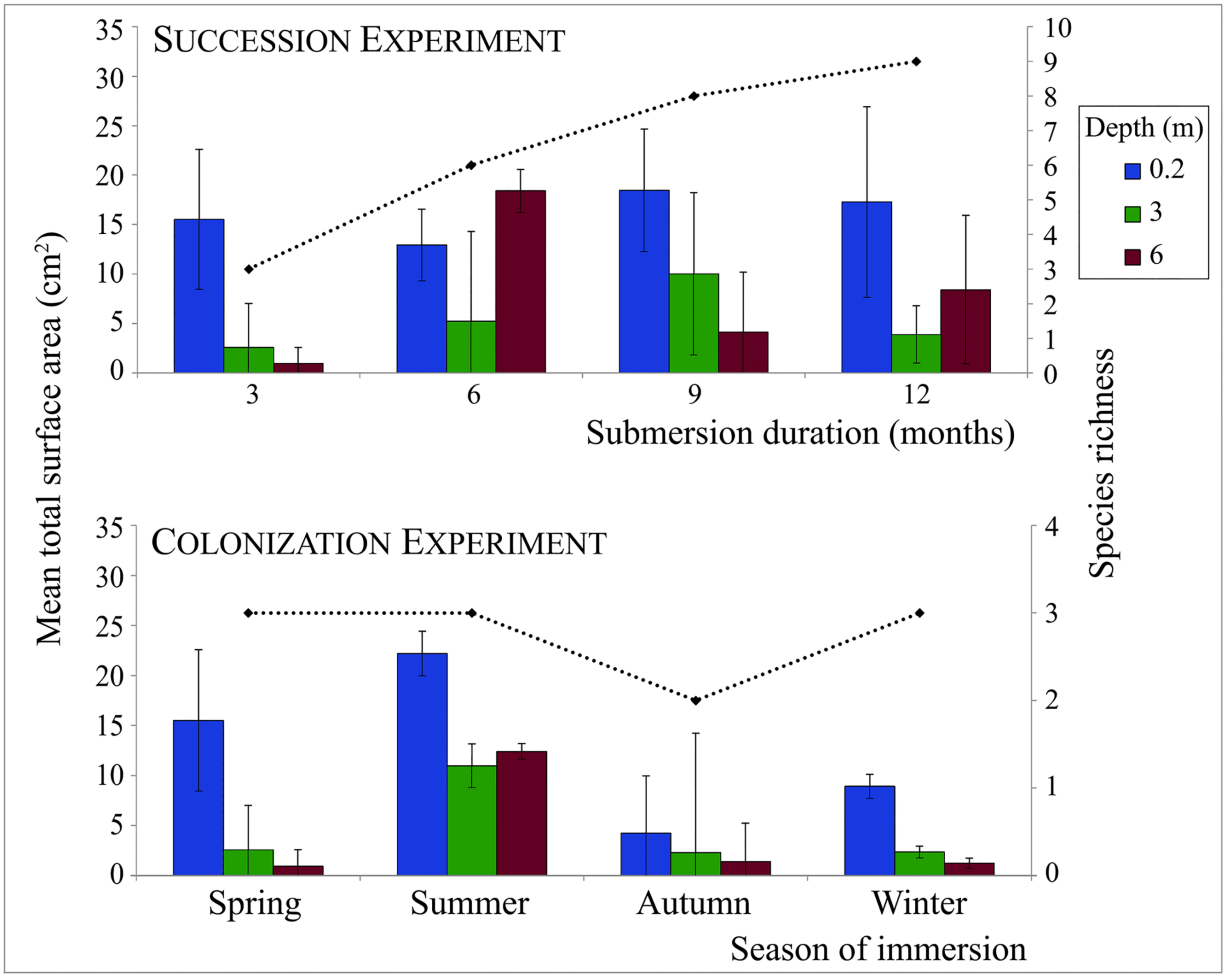
Variation in species richness (dashed line, total values) and surface cover (bars and error bars, mean ± standard deviation) occupied by fouling hydroids on the test panels during the succession and colonization experiments.

Fouling hydroids were present on the panels on all dates and at all depths during the succession experiment. An average of 9.8 cm^2^ (± 6.5) was occupied by hydroids on any of the 36 panels at any immersion time and depth, which represented about 5% (± 3.3%) of the available surface area. Considerable variation was observed in mean surface covered by fouling hydroids among panels (Fig 2); however, this variation was not statistically significant in relation to the submersion duration or to the depth of immersion (Table 1).

**Table 1:** Two-way ANOVA analyses of the effects of selected factors on the mean surface covered by fouling hydroids and species richness in the succession and colonization experiments.

**Succession experiment**					
*Mean surface covered by fouling hydroids*					
Source	DF	SS	MS	F	P
Submersion duration (S)	3	169.028	56.343	0.32	0.808
Depth (D)	2	739.078	369.539	2.13	0.141
S x D	6	497.204	82.867	0.48	0.818
Residual	24	4164.574	173.524		
Total	35	5569.884			
*Species richness*					
Source	DF	SS	MS	F	P
Submersion duration (S)	3	45.111	15.037	8.59	**0.001**
Depth (D)	2	16.056	8.029	4.59	0.121
S x D	6	5.056	0.843	0.48	0.816
Residual	24	42.000	1.750		
Total	35	108.222			
**Colonization experiment**					
*Mean surface covered by fouling hydroids*					
Source	DF	SS	MS	F	P
Season of immersion (S)	3	1565.525	521.842	4.17	**0.010**
Depth (D)	2	690.362	345.181	2.76	0.084
S x D	6	860.183	143.364	1.14	0.367
Residual	24	3005.042	125.210		
Total	35	6121.111			
*Species richness*					
Source	DF	SS	MS	F	P
Season of immersion (S)	3	1.639	0.546	0.94	0.438
Depth (D)	2	0.167	0.083	0.14	0.868
S x D	6	0.944	0.157	0.27	0.946
Residual	24	14.000	0.583		
Total	35	16.750			

Statistically significant results (P < 0.05) are shaded in grey. DF = degrees of freedom; SS = sum of squares; MS = mean squares; F = F statistic; P = probability value.

**Fig 3:**
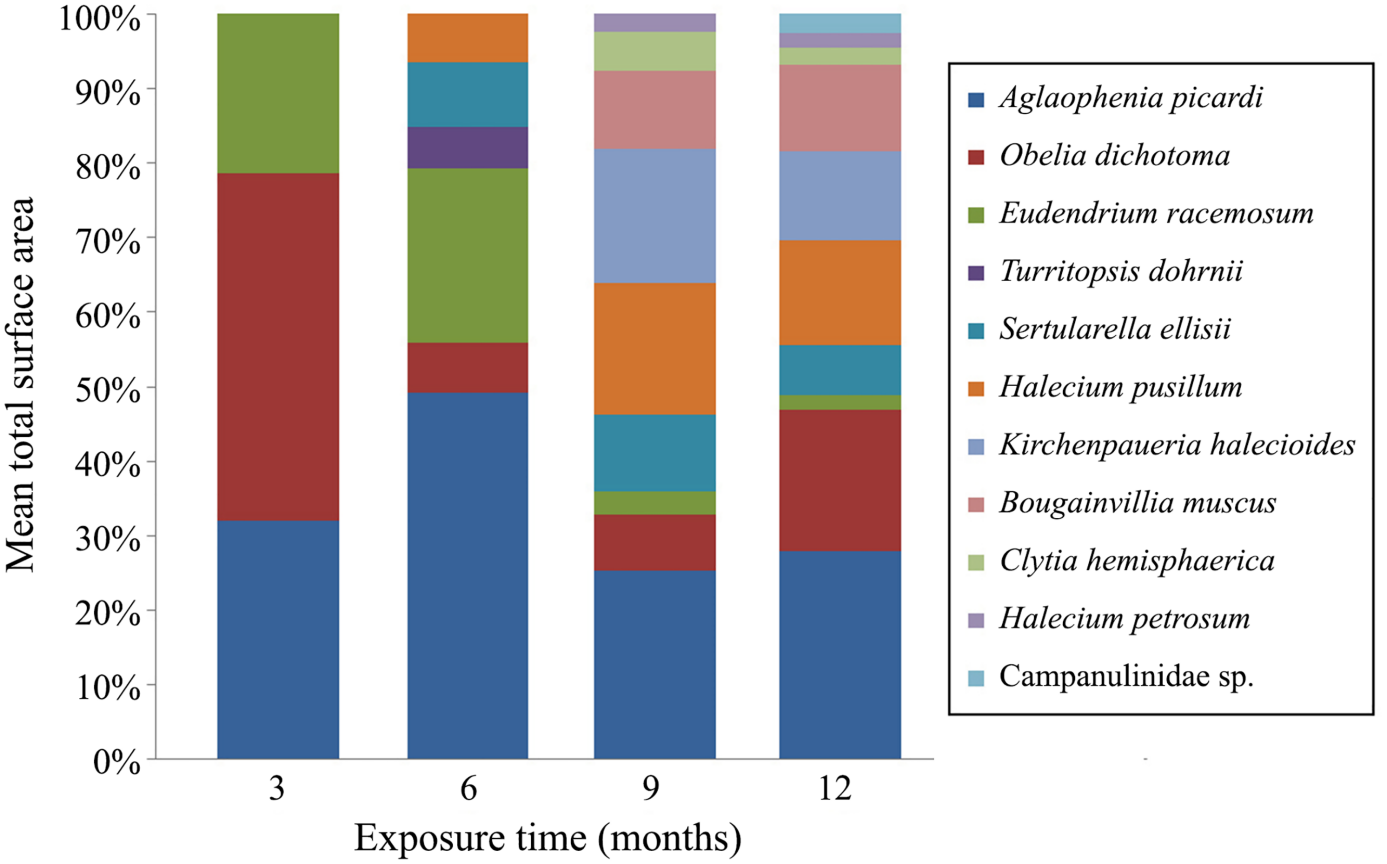
Percentage of mean surface cover for every species of fouling hydroid on the test panels during the succession experiment.

A total of 11 hydrozoan taxa were identified as part of the fouling community on the panels (Fig 3). Specimens in the family Campanulinidae could not be identified to species level due to the lack of reproductive structures and few polyps collected. The number of hydroid species increased significantly with duration underwater, from only 3 species after 3 months to 9 after 12 months. Species richness did not differ significantly on panels at different depths (Table 1).

**Fig 4:**
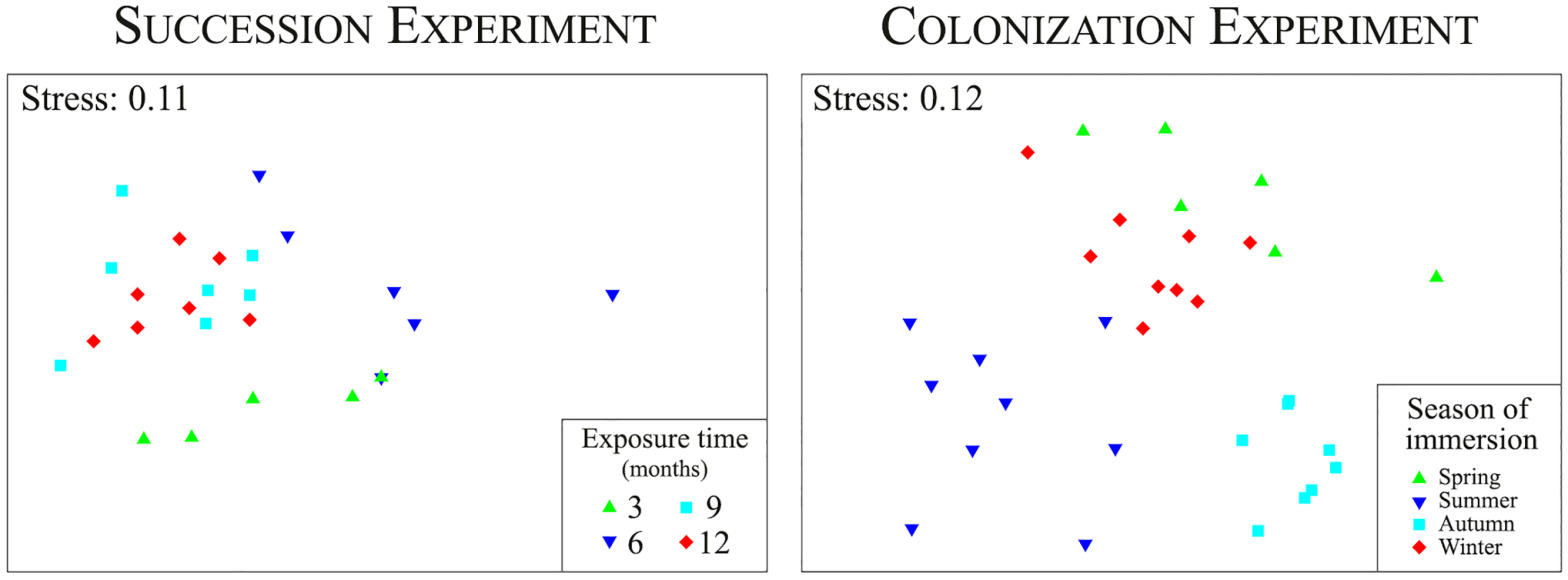
Non-metric multidimensional scaling (nMDS) plots based on Bray-Curtis similarity of surface covered by fouling hydroids in the succession and colonization experiments.

The hydrozoan species composition of the fouling assemblages changed with submersion duration, but the panel depth did not significantly affect the assemblages, as shown by the PERMANOVA analysis (Table 2). The nMDS diagram showed differences between the panels submerged for 3 months and those submerged for 9 and 12 months, which grouped together. The hydroid assemblages from 3- and 6-month panels overlapped little with those submerged for 9 and 12 months (Fig 4).

**Table 2:** PERMANOVA (permutational multivariate analysis of variance) results of tested factors on community composition based on surface cover of species in the succession and colonization experiments.

**Succession experiment**						
Source	DF	SS	MS	Pseudo-F	P (perm)	Unique perms
Submersion duration (S)	3	12061	4020.2	2.828	**0.018**	999
Depth (D)	2	9336.1	4668.1	2.284	0.109	998
S x D	6	13336	2222.6	1.564	0.122	996
Residual	16	22742	1421.4			
Total	27	62595				
**Colonization experiment**						
Source	DF	SS	MS	Pseudo-F	P (perm)	Unique perms
Season of immersion (S)	3	56220	18740	9.312	**0.001**	998
Depth (D)	2	3571.5	1785.7	0.887	0.592	997
S x D	6	16958	2826.4	1.404	0.059	997
Residual	20	40251	2012.6			
Total	31	121000				

Statistically significant results (P < 0.05) are shaded in grey. DF = degrees of freedom; SS = sum of squares; MS = mean squares; Pseudo-F = Pseudo-F statistic; P (perm) = probability after the permutations; Unique perms = permutations performed.

The SIMPER analysis showed that *Aglaophenia picardi* Svoboda, 1979, *Obelia dichotoma* (Linnaeus, 1758), and *Eudendrium racemosum* (Cavolini, 1785) characterized the assemblages on panels submerged for 3 and 6 months, while variations in the abundances of *Halecium pusillum* Sars, 1856, *Kirchenpaueria halecioides* (Alder, 1859), *Sertularella ellisii* (Deshayes & Milne Edwards, 1836), *O*. *dichotoma*, and *Bougainvillia muscus* (Allman, 1863) characterized assemblages on panels submerged for 9 and 12 months (Table 3). *Aglaophenia picardi*, *O*. *dichotoma*, and *E*. *racemosum* were the only species present on the panels recovered after 3 months. The surface occupied by these three species decreased over time as other species appeared on the panels (e.g., *Turritopsis dohrnii* (Weismann, 1883), *K*. *halecioides*, *S*. *ellisii* and *H*. *pusillum*). Later stages of succession had more species and more area covered by epibiotic colonies of *B*. *muscus*, *Clytia hemisphaerica* (Linnaeus, 1767), *Halecium petrosum* Stechow, 1919, *H*. *pusillum*, and Campanulinidae species. The most common and abundant species through all immersion times and depth levels were *A*. *picardi* and *O*. *dichotoma* (Fig 3).

**Table 3:** Average similarity (SIMPER) within fouling hydroid assemblages from panels with different submersion duration (succession experiment) and panels with different seasons of immersion (colonization experiment).

**Succession experiment**					
	Av. Surf.	Av. Sim.	Sim/ SD	Contrib %	Cum %
*Panels submerged for3 months*					
Average similarity: 66.67					
*Obelia dichotoma*	0.8	50	1.60	75	75
*Aglaophenia picardi*	0.6	16.67	0.58	25	100
*Panels submerged for 6 months*					
Average similarity: 49.17					
*Aglaophenia picardi*	0.67	39.17	1.38	79.66	79.6
*Eudendrium racemosum*	0.33	10	0.5	20.34	100
*Panels submerged for 9 months*					
Average similarity: 46.14					
*Halecium pusillum*	0.75	15.69	1.01	34.01	34.1
*Aglaophenia picardi*	0.63	13.11	1.03	28.41	62.4
*Obelia dichotoma*	0.75	11.88	0.74	25.76	88.2
*Sertularella ellisii*	0.25	2.86	0.38	6.19	94.4
*Panels submerged for 12 months*					
Average similarity: 61.49					
*Halecium pusillum*	0.89	19.99	1.66	32.51	32.5
*Obelia dichotoma*	0.78	14.35	1.02	23.33	55.8
*Bougainvillia muscus*	0.44	8.42	0.66	13.7	69.5
*Aglaophenia picardi*	0.44	7.87	0.66	12.79	82.3
*Clytia hemisphaerica*	0.44	6.17	0.49	10.04	92.4
**Colonization experiment**					
*Panels submerged from May to July 2013*					
Average similarity: 35.04					
*Obelia dichotoma*	5.89	26.37	1.68	75.27	75.3
*Aglaophenia picardi*	4.95	8.66	0.58	24.73	100
*Panels submerged from August to October 2013*					
Average similarity: 24.44					
*Eudendrium racemosum*	20.34	46.69	1.45	96.45	96.5
*Panels submerged from November 2013 to January 2014*					
Average similarity: 48.41					
*Kirchenpaueria halecioides*	7.84	23.39	1.67	95.72	95.7
*Panels submerged from February to April 2014*					
Average similarity: 53.90					
*Aglaophenia picardi*	1.86	33.2	1.29	61.59	61.6
*Obelia dichotoma*	0.75	15.67	0.8	29.07	90.7

Av. Surf. = average surface area covered; Av. Sim. = average similarity; Sim/SD = similarity to standard deviation ratio; Contrib % = percentage contribution; Cum. % = cumulative percentage contribution.

**Fig 5:**
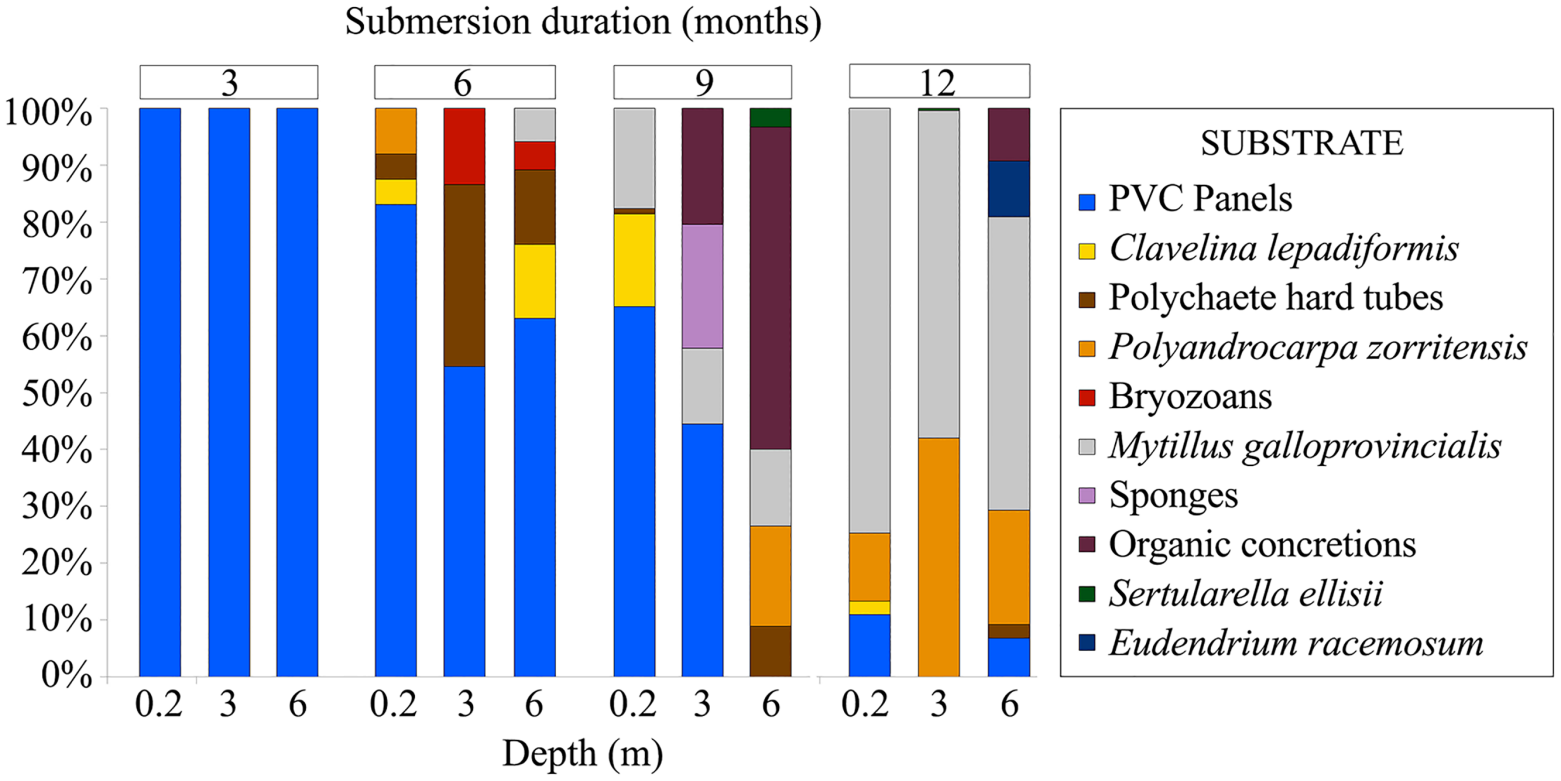
Substrate preference (percentage of occurrences per available substrate in each combination of depth and submersion duration) of the fouling hydroids in the succession experiment.

Substrate preferences of the fouling hydroids changed with the submersion duration of the panels (Fig 5). In the first 3 months of immersion, the only surface available for colonization was the artificial substrate of the PVC panels. After 6 months, new fouling biota appeared (polychaetes, tunicates, bryozoans, and mussels), which provided new available substrates for fouling hydrozoans. On the panels submerged for 9 and 12 months, the shells of fouling mussels (*Mytilus galloprovincialis* Lamarck, 1819) became the main substrate for the epibiotic species of hydroids, including many *H*. *pusillum* colonies. In later stages of succession, hydroids grew on other hydroids, especially *C*. *hemisphaerica* and Campanulinidae species, which used *S*. *ellisii* and the stem of *E*. *racemosum* as substrates.

Four species produced reproductive structures during the experiment: *A*. *picardi*, *O*. *dichotoma*, *C*. *hemisphaerica* and *B*. *muscus*. The last three species release medusae as part of their life cycle, with fertile colonies appearing on panels submerged for 3–12 months. The mean number of medusae produced per 100 cm^2^ reached maxima of 2.6 (±19.6) for *O*. *dichotoma*, 2.2 (±19.1) for *C*. *hemisphaerica*, and 0.2 (±1.7) for *B*. *muscus*. Numerous asexually-produced planktonic propagules were produced by *H*. *pusillum* (mean of 3.0 ± 21.3 per 100 cm^2^) mostly on the panels submerged for 9 and 12 months.

### Colonization experiment

The pioneer hydrozoan species on the panels were always a subset of those observed in the succession experiment. All the panels immersed for 3 months at different seasons of the year included hydrozoans as part of the colonizing fouling fauna. The mean surface covered by the hydroids differed significantly by season of immersion (Fig 2); however, depth did not have a significant effect on mean surface cover of fouling hydroids in the colonization experiment (Table 1).

In all, 7 hydroid species (*A*. *picardi*, *O*. *dichotoma*, *E*. *racemosum*, *S*. *ellisii*, *K*. *halecioides*, *H*. *petrosum*, and *B*. *muscus*) colonized the panels immersed for 3 months, although only 2 or 3 of these species grew simultaneously on any one panel. In fact, the number of fouling hydroid species observed in the colonization experiment did not differ significantly among depths or seasons (Table 1).

The season when the panels were immersed significantly affected the composition of fouling hydrozoan assemblages, while the depth of the panels did not significantly affect the colonization, as shown by the PERMANOVA (Table 2). The panels immersed during different seasons had clearly different fouling hydroid assemblages (Fig 4). The species characterizing the hydroid communities on the panels (i.e. those with highest percentage of cover and identified by the SIMPER analysis) were *A*. *picardi* and *O*. *dichotoma* in spring and winter, *E*. *racemosum* in summer, and *K*. *halecioides* in autumn (Table 3).

Fouling colonies of *A*. *picardi*, *K*. *halecioides* and *O*. *dichotoma* were producing reproductive structures (sessile gonophores in the first two species and gonothecae with medusa buds in the third) in the colonization experiment. Fertile colonies appeared on panels submerged in all seasons: *A*. *picardi* in spring and summer, *K*. *halecioides* in winter and spring, and *O*. *dichotoma* all year. The potential number of medusae released by colonies of *O*. *dichotoma* during the colonization experiment reached maximum means of 2.4 (±15.3) per 100 cm^2^ on the winter and spring panels, corresponding with the highest reproductive effort of the fouling hydroid colonies and contrasting with the lowest reproductive effort recorded in summer.

## Discussion

Hydrozoans represent a conspicuous component of the fouling assemblages in the studied aquaculture facility and are likely to regularly interact with cultured fish and shellfish. Their abilities to grow rapidly and to settle and re-settle on different surfaces allow hydroids to be among the first metazoans to colonize available substrates and ensure their presence during the entire process of succession [30–31]. The degree and outcomes of the interactions between fouling hydroids and fish, however, will change in time and space, in part modulated by the four main patterns emerging from our analysis: (i) increasing species richness as succession proceeded; (ii) changing species composition according to season and duration of exposure; (iii) progressively shifting substrate usage to include newly-created biotic surfaces; (iv) producing numerous planktonic propagules in all stages of succession and throughout the year.

The changes in hydroid composition and abundance follow the same patterns described for succession in natural hard-substrate communities, with certain species colonizing the substrate and, as succession proceeds, modifying the environment such that it becomes unsuitable for further recruitment, but facilitating settlement and development of a new group of species [32–33]. In the studied assemblages, the submersion duration was particularly important in determining the succession, through variation in larval abundance, arrival of new species, and biological interactions between biofoulers [34–35]. Successful hydroid biofoulers display different abundances and growth rates as succession progresses, reflecting their unique life history traits (e.g. production of planktonic stages, asexual reproduction, survival of the planulae), which in temperate regions are subjected to severe temporal fluctuations [36–37]. In fact, as for hydrozoans in natural communities, seasonal variation in environmental conditions affects the establishment, survival and growth of fouling hydroids [37–38] and, therefore, determines the composition and structure of biofouling assemblages and succession.

The observed increase in hydrozoan species with time (i.e., submersion duration) reflected the growing complexity of the fouling assemblages and the ability of hydroids to settle and grow on their competitors, capitalizing on the increased surface generated by other biofoulers (e.g., mussels, sponges, tunicates, bryozoans, polychaetes). Despite this, the observed fouling hydrozoans constituted only a very small portion (ca. 8%) of the total number of species (115) recorded from the surrounding coastal habitats [26]. More generally, fewer species are often encountered as biofoulers on artificial substrates compared to species-rich assemblages of natural habitats [39–41]. Which species colonized depended primarily on the season when the panels were immersed; specifically, different sets of species were observed on panels deployed in spring, summer, autumn, and winter. Experimental evidence suggests that both the season when a structure is immersed and the duration of submersion are more important than the type of substrate in structuring subtidal biofouling communities [42]. The season of immersion has been widely recognized as one of the most important factors modulating the outcome of succession in natural and artificial assemblages [34, 43–44]. As with the submersion duration, the time of the year when new substrates become available for colonization has a crucial influence on succession because of the seasonal differences of environmental conditions, reproduction, and growth patterns of fouling species, eventually affecting the pattern and rate of succession [34, 44–46]. Nevertheless, season may not determine the outcome in natural hard-bottom communities, because the stronger competitors eventually monopolize the available space despite initial differences in colonization, thus making the succession process partially predictable [33].

In contrast, the depths at which our test panels were submerged did not have a significant role in structuring the fouling hydrozoan assemblages. Although many physical factors change with depth and strong vertical zonation has been observed in natural and biofouling communities [47–49], most examples of depth-related vertical zonation come from depth differences larger than those studied here. Distinctly different communities are not usually found in the first 5–6 m from the surface on man-made structures [50]; the panels deployed in our study were assumed not to be subjected to highly different conditions of temperature, pressure, light, food, or nutrients. Therefore, a lack of vertical zonation in the analyzed biofouling assemblages was expected.

All of the hydrozoan species observed have been recorded previously from the surrounding hard-bottom benthic community [25–26], which can be considered the basic pool of colonizers. Furthermore, previously reported fouling hydroids in the area include *O*. *dichotoma* as well as species of *Aglaophenia*, *Sertularella*, and *Eudendrium* [39, 51]. In general, the species recorded here can be classified into two main groups: (i) ‘colonizers’ or ‘early successional species’ with tendencies to produce abundant asexual propagules and display rapid growth rates, and (ii) ‘mid- and late-successional species’, which tend to rely on efficient utilisation of resources to outcompete colonizers. The first group included *A*. *picardi*, *O*. *dichotoma*, *E*. *racemosum*, and *K*. *halecioides*, depending on the season. The second group was formed by epibiotic, less generalist species such as *H*. *pusillum*, *H*. *petrosum*, and Campanulinidae sp.

The best example of the pioneering and early successional hydrozoans recorded here may be *O*. *dichotoma*, a common and abundant species often encountered in disturbed sites and fouling communities. Contardo-Jara et al. [52] found it to be particularly abundant in disturbed communities growing on PVC panels in the southwestern Atlantic. It also has been reported as part of the biofouling assemblages of fish farms in the southwestern Pacific and northeastern Atlantic oceans, growing on coated and uncoated nets [5, 53]. It is a frequent species in harbours [40], where it often predominates over other fouling species [54], which may explain its ubiquity in the studied fouling assemblages. It is also one of the most common and abundant fouling hydrozoans on power stations and other artificial substrates along Italian coasts [55–57]. Blooms of *Obelia* spp. jellyfish occur along temperate coasts together with massive occurrence of hydroids [58]. *Obelia* species are frequent and widespread fouling organisms found abundantly virtually everywhere in aquaculture facilities: *Obelia longissima* (Pallas 1766) is common on Atlantic salmon cages and mussel nets at high latitudes in the Northern hemisphere [59–60], while *Obelia* spp. have been reported growing on finfish aquaculture cages in the Mediterranean [60], northeastern Pacific [61], and North Atlantic [62], in some cases contributing to problems due to occlusion of the net mesh aperture in the cages. *Obelia* spp. were also recorded growing on shellfish aquaculture facilities and on the shells of target species, as observed in the Eastern Mediterranean for mussels [63] and in the tropical southwestern Pacific for pearl oysters [64]. The high abundance of fouling *Obelia* spp. hydroids was suggested to be one of the factors potentially causing the observed high mortalities in shellfish aquaculture facilities, while the massive production of medusae by those hydroids substantially changed the quality of the nearby plankton community [65].

The dynamics of early successional species such as *Obelia* spp. contrast sharply with that of mid- and late-successional taxa such as *H*. *pusillum* or *H*. *petrosum*, which are smaller, slightly more specialized species commonly found growing on living substrates [66]. Other fouling hydrozoan species observed at the fish farm in Taranto also have been reported from aquaculture facilities around the world. As examples, eudendriid and bougainvilliid hydroids (including *B*. *muscus*) grow on nets containing scallops (*Pecten maximus* (Linnaeus, 1758)) or Atlantic salmon (*Salmo salar* Linnaeus, 1758) in the northern Atlantic and Pacific oceans [62, 67–68]; *Clytia* spp. are commonly recorded on culture tanks, nets, and PVC panels worldwide [62, 67, 69–70]; species of *Kirchenpaueria* and *Sertularella* may grow on ropes and nets of finfish and shellfish farms [60, 71].

Hydrozoan species that require the development of some structural elements prior to their settlement arrive after the rapidly growing, opportunistic colonizers. This implies a strong relationship between the development of the fouling community as a whole and the associated hydrozoan component during succession. During early succession almost 100% of hydrozoans found on the panel surface were growing directly on the PVC, but this percentage decreased sharply with immersion duration after the diversity and abundance of substrates on the panels increased. Eventually, some species (including *E*. *racemosum* and *S*. *ellisii*) became substrates for other hydrozoans, while other hydroids thrived by closely following the dynamics of their invertebrate substrates (e.g., mussels, ascidians, etc.). The latter is best exemplified by *H*. *pusillum* growing on the mussel *M*. *galloprovincialis* and the non-indigenous tunicate *Polyandrocarpa zorritensis* (Van Name, 1931). Because mussels and ascidians gradually covered the experimental panels, the fouling hydrozoan biota shifted towards epibiotic species that are able to colonize and survive on mussel shells and effectively take advantage of the newly available living space. The same occurred on panels submerged near the study area on which *M*. *galloprovincialis* eventually became very abundant, leading to a profound change in the number of other biofoulers [39]. *Mytilus galloprovincialis* is known to effectively deter fouling on its shell surface, which limits the available space for colonization by other biofoulers and creates specific conditions that permits settlement of only a few species (such as *H*. *pusillum*) on its shell [72].

Dispersive free-swimming medusae or asexual planktonic propagules were released by fouling hydroids into the water column during all the stages of succession and in all seasons. *Halecium pusillum* asexually produces complex, heterotrophic propagules adapted to pelagic life and released independently of the environmental conditions [66, 73]. The release of these propagules is common in the life cycle of some *Halecium* species and has been hypothesized to promote dispersal [74]. Dispersion through asexual propagules can be a very important demographic process in a wide array of taxa (e.g., sponges, tunicates, bryozoans, algae) [75] and is particularly important among benthic hydrozoans [31], especially to maintain populations of *H*. *pusillum* in the Mediterranean Sea [66]. The planktonic propagules of *H*. *pusillum* carry nematocysts [73] and could damage the skin and gills of fish in the cages.

The potential effects of pelagic cnidarians on the health of farmed fish in aquaculture facilities are beginning to be recognized through recent studies showing that abundances of stinging species are strongly correlated with gill and skin lesions, as well as fish mortality [12, 14, 76]. In the case of the facility we studied, the observed medusa production in *O*. *dichotoma*, *C*. *hemisphaerica*, and *B*. *muscus*, with colonies of *O*. *dichotoma* releasing particularly high numbers of medusae from early stages of colonization and throughout the entire succession, could easily generate damage associated with nematocyst discharge and venom injection. Jellyfish of *O*. *dichotoma*, *C*. *hemisphaerica* and *B*. *muscus* represent a potential problem for the farmed fish of Taranto. Jellyfish also have been suggested to be important as vectors of bacterial diseases for farmed fish [11] and some hydromedusae are intermediate hosts of fish parasites [77–78].

Altogether, the observed fouling hydroid assemblages may pose substantial problems for aquaculture in terms of fish health and increased production costs. In addition to medusae, actinula larvae and planktonic propagules, the benthic stages of fouling hydrozoans can directly harm the skin and gills of fish, as has been documented for *E*. *larynx* in Irish salmon farms [19]. Hydroid species also negatively affect shellfish and crustacean cultures by competing for food [69, 79–80], predating on larvae of the target species [20, 81], and inhibiting spat settlement [82]. More commonly, the presence of fouling hydroids has been linked to decreased water flow and increased net weight in fish cages [17, 62, 68]. Any of these negative impacts could occur in aquaculture facilities as the abundance of fouling hydrozoans increases. The common local biofouler *O*. *dichotoma*, for instance, has been linked to decreased water flow and increased net weight in cultures of the deep-sea scallop *Placopecten magellanicus* (Gmelin 1791) in the northwestern Atlantic [15] and is one of the parasite reservoirs for amoebic gill disease of cultured salmon in southwestern Pacific waters [83]. In the Mediterranean Sea, however, few estimates exist of the damage caused to finfish aquaculture by fouling hydrozoans [14] and, generally, no information is available on the agents causing mortality or disease of cultured fish. Thus, regular monitoring of the hydrozoan fouling assemblages is necessary to further increase understanding of their potential effects on the health of cultured fish and shellfish.

## Supporting information

S1 Table Temperature data for the Gulf of Taranto thoughout the study period. (XLSX)

S2 Table Standardized surface area covered by each hydrozoan species on each panel. (XLSX)
