# Correction: Successional dynamics of marine fouling hydroids (Cnidaria: Hydrozoa) at a finfish aquaculture facility in the Mediterranean Sea

**DOI:** 10.1371/journal.pone.0196883

**Published:** 2018-05-01

**Authors:** Luis Martell, Roberta Bracale, Steven A. Carrion, Adriana Giangrande, Jennifer E. Purcell, Marco Lezzi, Cinzia Gravili, Stefano Piraino, Ferdinando Boero

Dr. Adriana Giangrande should be included in the author byline. She should be listed as fourth author, and her affiliation is 2: Dipartimento di Scienze e Tecnologie Biologiche e Ambientali, Università del Salento, Lecce, Italy. The contributions of this author are as follows: Conceptualization, Investigation, Methodology, Resources.

The correct citation is: Martell L, Bracale R, Carrion SA, Giangrande A, Purcell JE, Lezzi M, et al. (2018) Successional dynamics of marine fouling hydroids (Cnidaria: Hydrozoa) at a finfish aquaculture facility in the Mediterranean Sea. PLoS ONE 13(4): e0195352. https://doi.org/10.1371/journal.pone.0195352

The following information is missing from the Funding section: This work was supported by the European Union’s project CERES (Climate Change and European Aquatic Resources) Horizon 2020 programme [678193 to SP].
